# Comparative impact of diverse regulatory loci on *Staphylococcus aureus* biofilm formation

**DOI:** 10.1002/mbo3.250

**Published:** 2015-03-21

**Authors:** Danielle N Atwood, Allister J Loughran, Ashleah P Courtney, Allison C Anthony, Daniel G Meeker, Horace J Spencer, Ravi Kr Gupta, Chia Y Lee, Karen E Beenken, Mark S Smeltzer

**Affiliations:** 1 Department of Microbiology and Immunology, University of Arkansas for Medical SciencesLittle Rock, Arkansas; 2 Department of Biostatistics, University of Arkansas for Medical SciencesLittle Rock, Arkansas; 3 Department of Orthopaedic Surgery, University of Arkansas for Medical SciencesLittle Rock, Arkansas; 4 Department of Pathology, University of Arkansas for Medical SciencesLittle Rock, Arkansas

**Keywords:** Biofilm, protease, regulation, *sarA*, *Staphylococcus aureus*

## Abstract

The relative impact of 23 mutations on biofilm formation was evaluated in the USA300, methicillin-resistant strain LAC. Mutation of *sarA*, *atl*, *codY*, *rsbU*, and *sigB* limited biofilm formation in comparison to the parent strain, but the limitation imposed by mutation of *sarA* was greater than that imposed by mutation of any of these other genes. The reduced biofilm formation of all mutants other than the *atl* mutant was correlated with increased levels of extracellular proteases. Mutation of *fur*- and *mgrA*-enhanced biofilm formation but in LAC had no impact on protease activity, nuclease activity, or accumulation of the polysaccharide intercellular adhesin (PIA). The increased capacity of these mutants to form a biofilm was reversed by mutation of *sarA*, and this was correlated with increased protease production. Mutation of *sarA*, *mgrA,* and *sigB* had the same phenotypic effect in the methicillin-sensitive strain UAMS-1, but mutation of *codY* increased rather than decreased biofilm formation. As with the UAMS-1 *mgrA* mutant, this was correlated with increased production of PIA. Examination of four additional clinical isolates suggests that the differential impact of *codY* on biofilm formation may be a conserved characteristic of methicillin-resistant versus methicillin-sensitive strains.

## Introduction

Many forms of *Staphylococcus aureus* infection are characterized by formation of a bacterial biofilm, the presence of which confers a therapeutically relevant level of intrinsic resistance to both host defenses and conventional antibiotics (Brady et al. 2008; Lewis 2008; Trotonda et al. 2008; Bjarnsholt et al. 2013). Among these are infections of bone and indwelling orthopedic devices, and given our specific interest in these infections, we have focused much of our effort on identifying factors that contribute to *S. aureus* biofilm formation (Tsang et al. 2008; Beenken et al. 2012, 2014; Cassat et al. 2013). Our results, as well as those from other laboratories, have led us to place a primary emphasis on the staphylococcal accessory regulator locus (*sarA*), mutation of which limits *S. aureus* biofilm formation to a degree that can be correlated with increased antibiotic susceptibility and an improved therapeutic outcome in relevant murine and rabbit models (Beenken et al. 2003; Valle et al. 2003; Weiss et al. 2009a,b; Abdelhady et al. 2014). However, *sarA* is part of a complex and highly interactive regulatory circuit that includes many other loci implicated in biofilm formation (Priest et al. 2012; Ibarra et al. 2013). This brings up two important questions, the first being whether other regulatory loci offer therapeutic potential comparable to or even greater than *sarA*, and the second being whether the functional status of other regulatory loci has the potential to compromise therapeutic strategies targeting *sarA*.

It is impossible to answer these questions because no comprehensive direct comparisons have been made under consistent experimental conditions. Indeed, there are reports that are directly contradictory by comparison to each other. For example, Tu Quoc et al. (2007) found that mutation of *mgrA* or *codY* limited biofilm formation, while other reports concluded that mutation of these same loci has the opposite effect (Majerczyk et al. 2008; Trotonda et al. 2008). One possible explanation for such disparate results is the use of different *S. aureus* strains, which is understandable, and in fact necessary, from a therapeutic point of view, particularly given the genetic and phenotypic diversity that exists among contemporary clinical isolates (Cassat et al. 2006; Wang et al. 2007; Klein et al. 2013). It has been suggested that methicillin resistance itself has a direct impact on the mechanism of biofilm formation, with methicillin-resistant strains relying primarily on surface proteins, most notably FnbA and FnbB, and methicillin-sensitive strains relying more heavily on the polysaccharide intercellular adhesin (PIA) (Pozzi et al. 2012).

It is also possible that such contradictory reports are due to the use of different in vitro methods of testing biofilm formation. Two primary examples include the medium used to assess biofilm formation and whether the substrate is first coated with human plasma proteins, the latter reflecting the fact that even abiotic medical implants are rapidly coated with host proteins after implantation (Francois et al. 1996). The in vitro assays that led to our initial focus on *sarA* employed tryptic soy broth (TSB) supplemented with both salt and glucose as well as a plasma-coated substrate (Beenken et al. 2003). Subsequent studies have confirmed that the phenotypes we observed under these conditions translate to a reduced capacity to form a biofilm in vivo (Weiss et al. 2009b) and a reduced capacity to cause hematogenous bone and joint infection (Zielinska et al. 2012). Nevertheless, it remains important to consider alternative assay conditions if for no other reason than to clarify discrepancies in the literature. Thus, we compared the relative capacity of 23 mutants to form a biofilm in vitro under different conditions. Primary experiments were done with the USA300 methicillin-resistant strain LAC and expanded to additional clinical isolates including the methicillin-sensitive strain UAMS-1. We also investigated the mechanistic basis for mutations correlated with an altered biofilm phenotype.

## Experimental Procedures

### Generation of primary mutants

Regulatory mutants generated in the plasmid cured JE2 derivative of the USA300, methicillin-resistant strain LAC (Fey et al. 2013) were obtained from the Nebraska Transposon Mutant Library (NTML) through the Network on Antimicrobial Resistance in *S. aureus* (NARSA, now available from BEI Resources, Manassas, VA, http://www.beiresources.org). To ensure consistency with our previous studies, and because the NTML consists of primary mutants that have not been characterized beyond their transposon insertion sites, each mutation was first transduced into the derivative of LAC and its isogenic *sarA* mutant employed in our previous studies (Zielinska et al. 2011). To generate the NTML, JE2 was cured of both its larger plasmid conferring resistance to erythromycin and its smaller cryptic plasmid (Fey et al. 2013), while the derivative of LAC we employ was cured only of the larger plasmid (Wormann et al. 2011). This allowed erythromycin selection of transductants, with confirmation subsequently obtained by PCR analysis of the targeted gene (data not shown) and by comparison of *Eco*RI-digested genomic DNA, which confirmed the presence of the small cryptic plasmid in the LAC recipients but not in the JE2 donors (Fig. S1). However, analysis of a subset of strains using our standard assay conditions (Beenken et al. 2003) demonstrated that the impact of individual mutations on biofilm formation was consistent in JE2 and our derivative of LAC (Fig. S1).

We also examined *codY*, *mgrA*, and *sigB* mutants generated in the MSSA osteomyelitis isolate UAMS-1, isogenic *sarA* mutants generated in both LAC and UAMS-1, and an isogenic mutant of LAC unable to produce all extracellular proteases other than those encoded by the *spl* operon (Beenken et al. 2003, 2014; Zielinska et al. 2011, 2012). This was necessitated by the fact that the *spl* mutation is defined by resistance to erythromycin, thus precluding the ability to use our LAC derivative unable to produce any extracellular protease (Zielinska et al. 2011) as a transduction recipient. However, previous studies confirmed that biofilm formation is comparable in LAC *sarA* mutants unable to produce any extracellular protease versus those that retain the capacity to produce only the *spl*-encoded proteases (Loughran et al. 2014). Phage-mediated transduction was also used to generate *codY* mutants in each of four additional clinical isolates. However, because these strains were resistant to erythromycin, and because all of the mutants obtained from the NTML are defined by erythromycin resistance, it was first necessary to exchange the erythromycin resistance cassette in JE2 to an alternative antibiotic resistance cassette (Bose et al. 2013). All mutations, and the identity of the recipient strain, were confirmed by PCR of the targeted gene and additional genes and/or mutations that define each recipient strain (data not shown). Mutants were then maintained at −80°C in TSB containing 25% (v/v) glycerol.

### Genetic complementation

Construction of an *rsbU* complementation plasmid was done by PCR amplification of the *rsbU* open reading frame (ORF) together with 556 bp of upstream DNA (forward oligonucleotide primer: GCGAAAATACCGACACATGTAG; reverse primer: GGGTTTTGAAGCTTTAAAATTGCTTC). The amplification product was cloned into the pCR2.1 TOPO vector (Invitrogen, Grand Island, NY) and transformed into Z-Competent *Escherichia coli* cells (Zymo Research Corp., Irvine, CA). After verification by DNA sequencing (data not shown), the plasmid was digested with *Eco*RI (New England Biolabs, Ipswitch, MA) and the insert ligated into the *E. coli-S. aureus* shuttle vector pLI50 (Blevins et al. 1999).

Construction of the *sigB* complementation plasmid was done by PCR amplification using a forward primer with an *Nde*I cut site (GGGCATATGGCGAAATAATGGCGAAAG) and a reverse primer that included a *Bam*HI cut site (CCCGGATCCCGTATCATTAATAAACAAATTC). The amplification product was ligated into pCR2.1, verified as described above, and the insert cloned into the shuttle vector pOS1 (Bubeck Wardenburg et al. 2006) such that expression of *sigB* was under the control of the lipoprotein diacylglycerol transferase promoter (pOS1-p*lgt*) (Torres et al. 2010). Amplification of *rsbU* and *sigB* was done using genomic DNA from the USA300 strain LAC as template.

The *mgrA* complementation plasmid was generated by PCR using a forward primer containing a *Hind*III restriction site and an N-terminal 6XHis tag (GGATCCAAGCTTATGCATCATCACCATCACCATGGATCTGATCAACATAATTTAAAAGAACAGCTATGC), the latter being added for purposes outside the scope of the experiments reported here, together with a reverse primer containing a *Hind*III restriction site (GGATCCAAGCTTTTATTTTTCCTTTGTTTCATCAAATGCATGAATGAC). The amplification product was cloned into the shuttle vector pLL48 under the control of an isopropyl β-D-1-thiogalactopyranoside (IPTG)-inducible promoter. Specifically, pLL48 was generated by cloning P*spac*-*lacI* promoter from pCL15 into pLL47 plasmid (Luong and Lee 2006; Luong et al. 2011). In this case, amplification was done using genomic DNA from the *S. aureus* strain Newman. Induction was done using 1 mmol/L IPTG.

The plasmid constructs used to complement the *atl, codY, fur,* and *sarA* mutations were all described previously (Blevins et al. 1999; Torres et al. 2010; Luong et al. 2011; Bose et al. 2012). Where appropriate, complementation plasmids were first used to transform the *S. aureus* strain RN4220 by electroporation. Once in *S. aureus*, plasmids were then introduced into the appropriate strains by phage-mediated transduction. Complemented strains were also maintained at −80°C in TSB containing 25% (v/v) glycerol. For each experiment, strains under study were retrieved from cold storage by plating on tryptic soy agar (TSA) with appropriate antibiotic selection. Antibiotics were used at the following concentrations: erythromycin, 10 *μ*g mL^−1^; tetracycline, 5 *μ*g mL^−1^; kanamycin, 50 *μ*g mL^−1^; neomycin, 50 *μ*g mL^−1^, spectinomycin, 1000 *μ*g mL^−1^; chloramphenicol, 10 *μ*g mL^−1^. Kanamycin and neomycin were always used together to avoid selection of spontaneously resistant strains.

### Assessment of biofilm formation

Biofilm formation was assessed in vitro using a microtiter plate assay. To explore the impact of different assay conditions, the medium consisted of TSB with and without supplementation with 3% sodium chloride and 0.5% glucose (biofilm medium, BFM), while the substrate was used with and without coating with 20% human plasma as previously described (Beenken et al. 2003). Briefly, bacterial cultures were grown at 37°C to stationary phase (16 h) in TSB or BFM with antibiotics when appropriate. Cultures were standardized to an OD_560_ = 0.05 in the appropriate test medium (TSB or BFM) without antibiotics. IPTG (1 mmol/L) or Dispersin B (Kane Biotech Inc, Winnipeg, Manitoba, Canada, 5 *μ*mol/L) was included as appropriate. Wells of a 96-well microtiter plate were then inoculated with 200 *μ*L and incubated at 37°C for 24 h, at which point they were washed three times with 200 *μ*L PBS, fixed with 200 *μ*L 100% EtOH, stained with 200 *μ*L crystal violet, and washed three times with 200 *μ*L PBS. Stain was then eluted with 150 *μ*L 100% EtOH for 10 min before diluting the eluent with an equal volume of PBS. Absorbance was measured using a BioTek Synergy 2 microplate reader (BioTek Instruments, Winooski, VT). For mixed culture biofilm assays with LAC, UAMS-1, and their *sarA* mutants, each strain was grown overnight in BFM, standardized as described above, and mixed in equal volumes prior to inoculation of the wells. All assays were performed using at least two biological replicates, each containing a minimum of three experimental replicates.

### Western blotting

SarA production was assessed using whole-cell lysates prepared from stationary phase cells and a rabbit polyclonal anti-SarA IgG antibody as previously described (Blevins et al. 1999). Secondary antibody was horseradish peroxidase (HRP)-conjugated goat anti-rabbit IgG (Sigma Chemical Co., St Louis, MO). Blots were performed in triplicate using different biological replicates. Blots were developed with SuperSignal West Femto Chemiluminescent Substrate (Thermo Scientific, Rockford, IL) and quantified using a Bio-Rad ChemiDocMP Imaging System and Image Lab Software (Bio-Rad Laboratories, Inc., Hercules, CA).

### Protease activity

Protease activity was assessed in standardized samples of cell-free supernatant from stationary phase (16 h) cultures grown without antibiotics using a Protease Fluorescent Detection Kit (Sigma Chemical Co.) as previously described (Zielinska et al. 2012). Results are reported as relative fluorescence units and represent at least two biological replicates, each of which included four experimental replicates.

### Nuclease activity

Nuclease activity was assessed using a fluorescence resonance energy transfer (FRET)-based assay as previously described (Beenken et al. 2012). Briefly, 25 *μ*L sterilized, standardized supernatants from stationary phase cultures (16 h) grown without antibiotic selection were mixed with an equal volume of FRET substrate (5′-/5HEX/CCCCGGATCCACCCC/3BHQ_2/-3′; Integrated DNA Technologies, Coralville, IA) diluted to 2 *μ*mol/L in buffer consisting of 20 mmol/L Tris, pH 8.0, and 10 mmol/L CaCl_2_. Results were assessed after 5 min at 30°C using an excitation wavelength of 530 nm and an emission wavelength of 590 nm. Results are reported as relative fluorescence units. Nuclease activity was also assessed using D'NASE Test Agar (REMEL, Lenexa, KS) (Tsang et al. 2008).

### PIA immunoblot

Production of the polysaccharide intercellular adhesion (PIA) was assessed as previously described with slight modifications (Beenken et al. 2004). Specifically, cultures were grown overnight in TSB supplemented with 3.0% sodium chloride and 0.5% glucose and antibiotics as appropriate. After standardization to OD_660_ = 5.0, cells were harvested by centrifugation and resuspended in 60 *μ*L 0.5 mol/L EDTA. Cell suspensions were boiled at 105°C for 8 min followed by centrifugation. Forty microliters of the supernatant was then incubated for 30 min with 5 *μ*L proteinase K (10 mg per mL) at 48°C to reduce nonspecific background levels. Twenty microliter of Tris-buffered saline (20 mmol/L Tris-HCl, 150 mmol/L NaCl [pH 7.4]) was added to the samples, which were then stored at −20°C. For analysis, 20 *μ*L of this sample was mixed with 60 *μ*L TBS. Using a BIO-dot microfiltration apparatus (Bio-Rad Laboratories, Inc.), 50 *μ*L was spotted onto a nylon membrane presoaked with TBS (Roche Diagnostics Corp., Indianapolis, IN). Each well was then rinsed with 200 *μ*L tris-buffered saline (TBS). The membrane was then removed, dried, and blocked in 0.5% skim milk overnight at 4°C. PIA production was assessed using anti-PIA antiserum (kindly provided by Michael Otto, National Institute of Allergy and Infectious Disease) diluted 1:500 in 0.5% skim milk. Primary antibody was detected using HRP-conjugated goat anti-rabbit IgG secondary antibody (Sigma Chemical Co.). Blots were developed and quantified as described above after subtracting the background observed with a UAMS-1 *ica* mutant.

### Statistical analysis

Statistical comparisons were done using the unpaired *t*-test or where appropriate one-way analysis of variance with Tukey's Multiple Comparison Test. Statistical analysis was done using GraphPad Prism 5.0 (La Jolla, CA).

## Results and Discussion

### Comparison of different assay conditions

**Figure 1:**
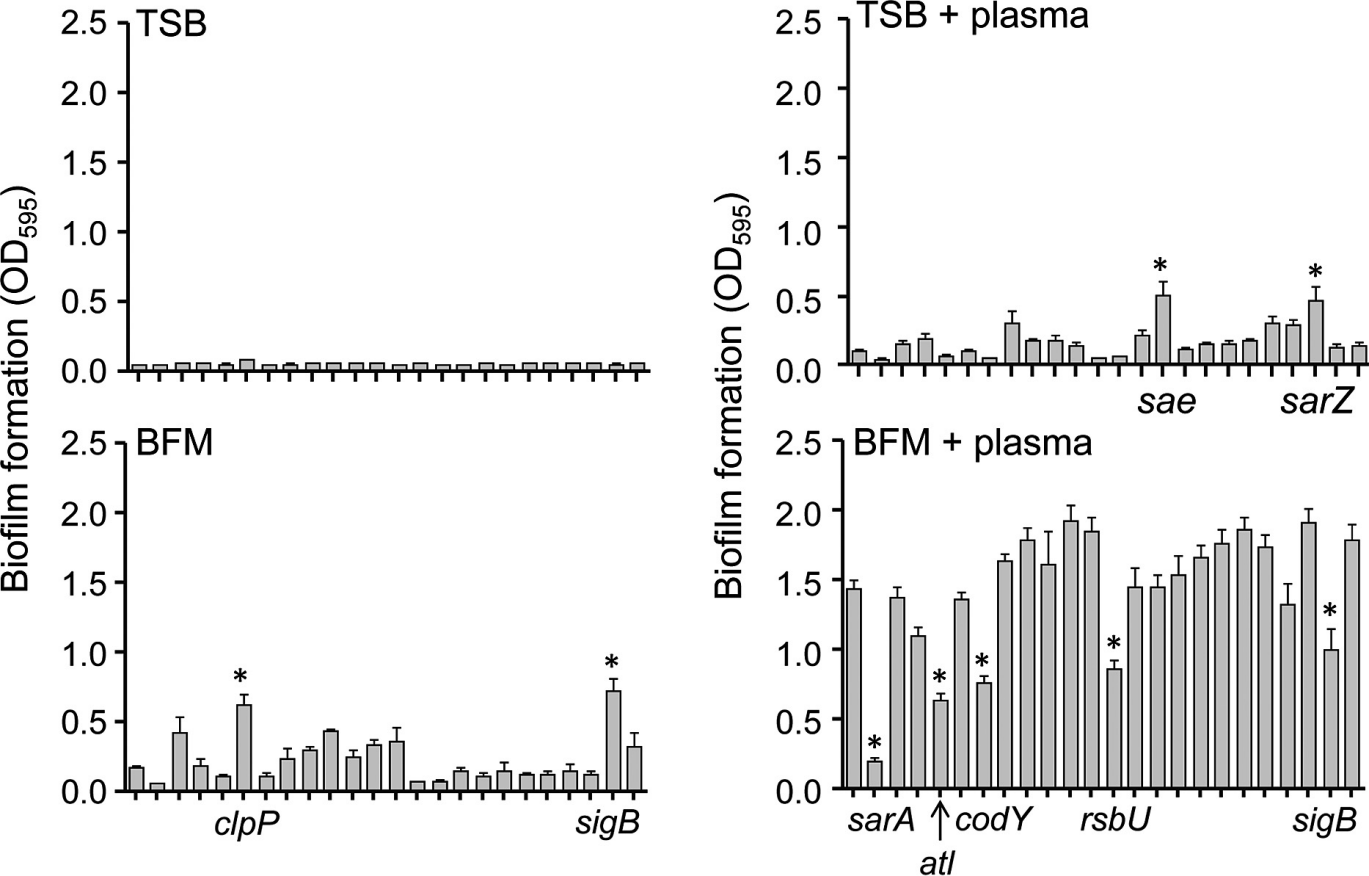
Biofilm phenotypes as a function of assay conditions. Biofilm formation was assessed in LAC and its isogenic mutants under four different assay conditions. To allow direct comparisons between conditions, the results shown in all panels are shown as raw data and represent the average ± standard error of the mean (SEM) from a minimum of two experiments, each of which was repeated with at least three replicates. Mutants that exhibit a statistically significant difference under each assay condition (asterisk; *P* < 0.05) are indicated in each panel. For every individual strain, including the *sarA* mutant, the results observed with BFM and plasma coating were statistically significant by comparison to the same strain assayed under all other conditions. Overall order in all panels is LAC followed by isogenic strains with mutations in *sarA*, *agr*, *arl*, *atl*, *clpP*, *codY*, *fur*, *lyt*, *mgrA*, *msa*, *rot*, *rsbU*, *rsr*, *sae*, *sarS*, *sarT*, *sarU*, *sarV*, *sarX*, *sarY*, *sarZ*, *sigB*, and *srr*.

LAC mutants were generated by phage-mediated transduction from JE2 donor strains obtained from the NTML (Fig. S1). A microtiter plate assay was then used to assess the relative capacity of these mutants to form a biofilm under different assay conditions. Neither LAC nor any of its regulatory mutants formed a biofilm when the assay was done in TSB without media supplementation and without plasma coating of the substrate (Fig.1). A statistically significant increase was observed with two mutants (*saeRS* and *sarZ*) when the assay was done in TSB with plasma coating but without media supplementation, as well as two different mutants (*clpP* and *sigB*) when the assay was done using uncoated plates and TSB supplemented with NaCl and glucose (BFM). However, under all three of these experimental conditions, biofilm formation was extremely limited by comparison to the results observed when the assay was performed using BFM and the substrate was coated with human plasma (Fig.1). In fact, biofilm formation was significantly increased under these conditions in every mutant, including the *sarA* mutant, by comparison to the same strain examined under all other assay conditions (Fig.1). Our original studies identifying *sarA* as a primary mediator of biofilm formation were done using BFM and a plasma-coated substrate, and subsequent studies confirmed its importance under in vivo conditions, thus suggesting that these in vitro conditions accurately reflect the likelihood of in vivo relevance (Weiss et al. 2009a,b; Beenken et al. 2010, 2014; Zielinska et al. 2012). Additionally, indwelling medical devices are rapidly coated with host proteins (Steckelberg and Osmon 1994; Gotz 2002), and it has been demonstrated that biofilm-associated bacteria encounter unique growth conditions that include increased osmolarity (Prigent-Combaret et al. 1999), both of which provide further support for the hypothesis that, by comparison to the other assay conditions we examined, the use of BFM and a plasma-coated substrate is more likely to reflect in vivo relevance. Thus, we employed these assay conditions in all subsequent experiments.

### Relative impact of regulatory mutations

**Figure 2:**
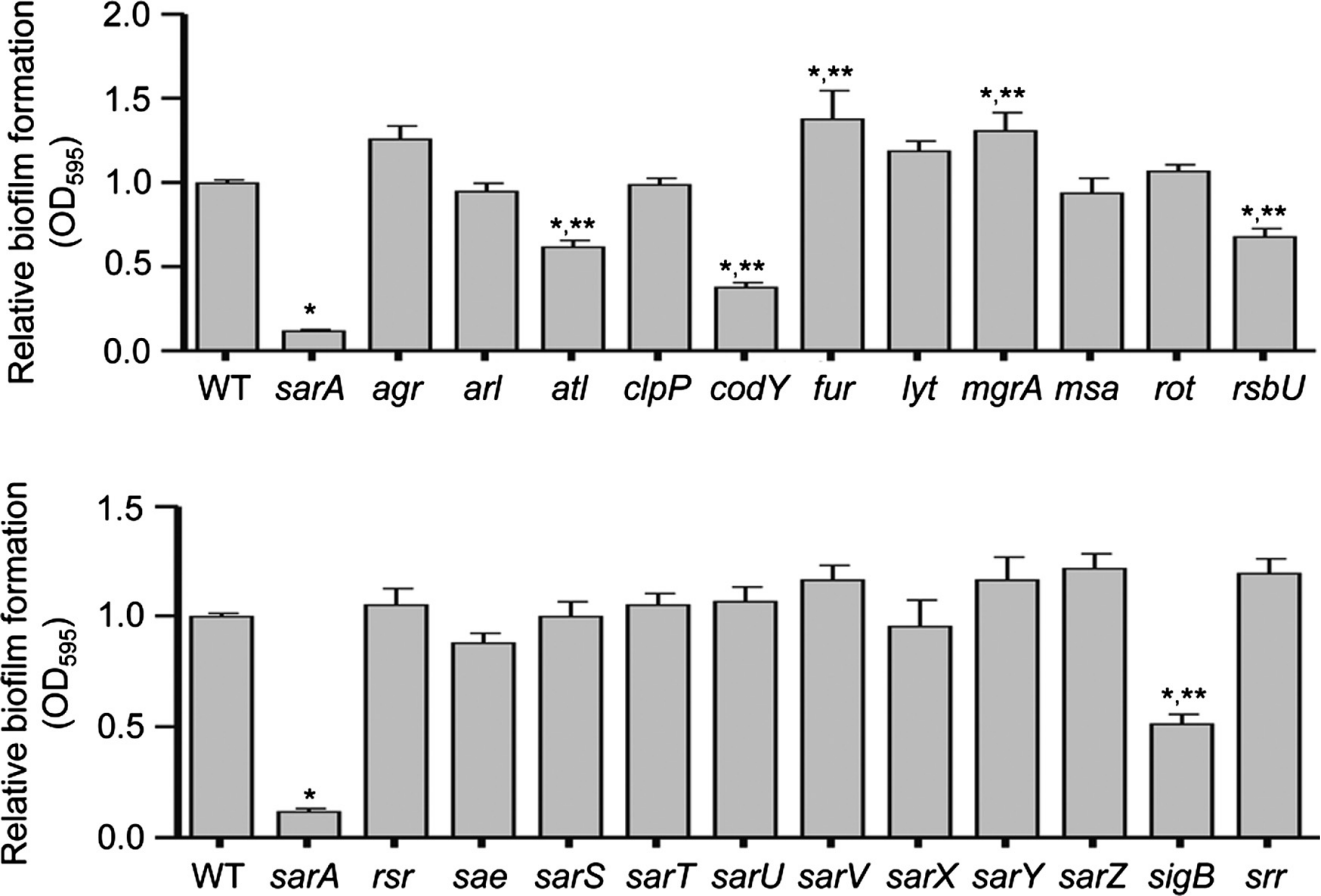
Relative impact of *Staphylococcus aureus* regulatory loci on biofilm formation in vitro. Biofilm formation was assessed in LAC (WT) and its isogenic regulatory mutants using a microtiter plate assay with BFM and plasma coating of the substrate. Results shown represent the average ± SEM from a minimum of six experiments, each of which was repeated with at least three technical replicates. Single asterisk indicates statistical significance by comparison to the parent strain (*P* < 0.05). Double asterisks indicate statistical significance by comparison to the isogenic *sarA* mutant (*P* < 0.05).

**Figure 3:**
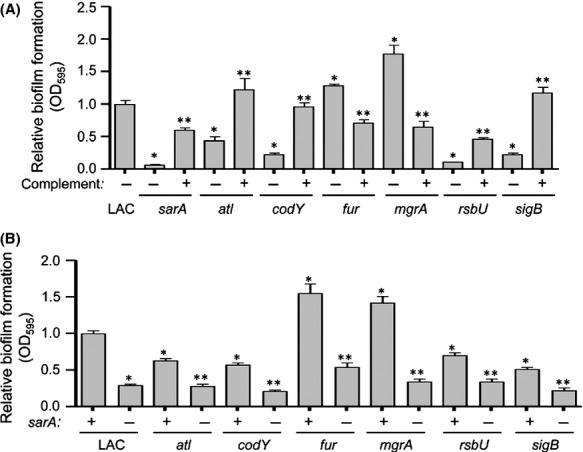
Relative impact of *sarA* versus other regulatory loci in LAC. (A) Biofilm formation was assessed in each regulatory mutant found to have a significant impact on biofilm formation with (+) and without (−) plasmid-based genetic complementation. Results shown represent the average ± SEM from a minimum of two experiments, each of which was repeated with at least three replicates. Single asterisk indicates that the results observed with the indicated mutant were significantly different from those observed with the LAC parent strain (*P* < 0.05). Double asterisks indicate that the results observed with the complemented strain were significantly different by comparison to those observed with the uncomplemented isogenic mutant (*P* < 0.05). (B) Biofilm formation was assessed in each regulatory mutant found to have a significant impact on biofilm formation with (−) and without (+) concomitant mutation of *sarA*. Single asterisk indicates statistical significance by comparison to the LAC parent strain (*P* < 0.05). Double asterisks indicate significance of the double mutant relative to the corresponding isogenic single mutant (*P* < 0.05).

We examined the biofilm phenotype of LAC and each of 22 regulatory mutants and an *atl* mutant using our optimized assay conditions. While not a regulatory element, Atl has been shown to play a critical role in the initial attachment stage of biofilm formation and the subsequent release of extracellular DNA (eDNA) further enhancing the process (Houston et al. 2011). As such, we felt it was necessary to include Atl in our comparative studies. Comparisons included a minimum of six biological replicates per strain, each of which included at least three experimental replicates. To make the biological replicates comparable to one another, the results observed with LAC were set to 1.0, with the results observed with each regulatory mutant shown relative to this value. Results from all replicates were then combined for statistical analysis. These studies identified seven mutants in which the capacity to form a biofilm was significantly different from that observed in the LAC parent strain (Fig.2). Five of these (*sarA*, *atl*, *codY*, *rsbU*, and *sigB*) had a reduced capacity to form a biofilm, while two (*fur* and *mgrA*) had an increased capacity to form a biofilm (Fig.2). The cause-and-effect relationship between all mutations and their biofilm phenotypes was confirmed by genetic complementation (Fig.3).

Although *atl*, *codY*, *rsbU,* and *sigB* mutants exhibited a decreased capacity to form a biofilm by comparison to the parent strain, they also exhibited a significantly increased capacity to form a biofilm by comparison to the isogenic *sarA* mutant (Fig.2). The fact that mutation of *sarA* had a greater impact on biofilm formation than mutation of these other genes was confirmed by demonstrating that concomitant mutation of *sarA* reduced biofilm formation still further in all of these mutants (Fig.3). Concomitant mutation of *sarA* also reversed the increased biofilm formation observed in the *fur* and *mgrA* mutants (Fig.3), thus confirming that the impact of mutating *sarA* is epistatic to that of mutating these other regulatory loci.

### Impact of regulatory mutations on accumulation of SarA

**Figure 4:**
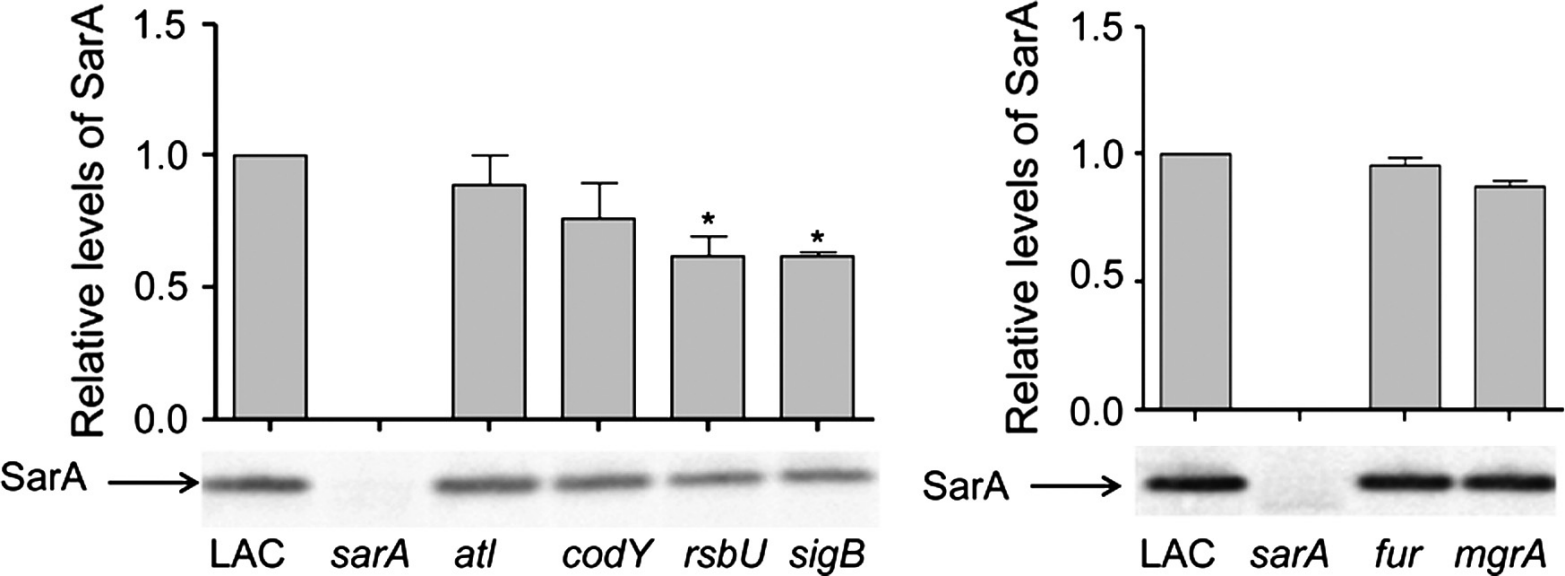
Impact of LAC regulatory mutations on accumulation of SarA. Relative amounts of SarA were assessed by western blot. Graphs illustrate quantitative results from three separate blots. Single asterisk indicates statistical significance by comparison to the LAC parent strain (*P* < 0.05).

Eliminating the production of an effector protein like SarA typically has a greater phenotypic impact than mutation of genes that modulate the production or activity of that effector protein. One explanation for the intermediate impact of mutating *atl*, *codY*, *rsbU,* and *sigB* on biofilm formation is that mutation of these loci limits, but does not eliminate, the production of SarA itself. The only mutations found to have a statistically significant impact in this regard were the *rsbU* and *sigB* mutations (Fig.4). This is consistent with the current *S. aureus* regulatory paradigm indicating that RsbU is a positive regulator of SigB, and SigB an activator of *sarA* expression (Bischoff et al. 2001; Cheung et al. 2008; Pane-Farre et al. 2009). This suggests that the impact of these loci is likely to be mediated, at least in part, via a *sarA*-dependent pathway, while that of *atl* and *codY* is mediated via a *sarA*-independent pathway. Similarly, mutation of *fur* or *mgrA* had no impact on the accumulation of SarA (Fig.4).

### Impact of extracellular protease production on biofilm formation

**Figure 5:**
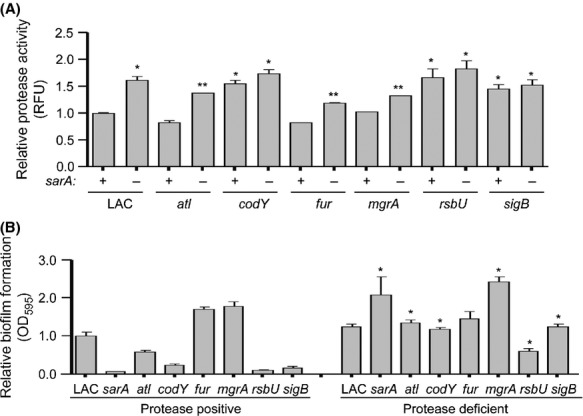
Impact of extracellular proteases in LAC. (A) Total protease activity was assessed in LAC mutants with (−) and without (+) concomitant mutation of *sarA*. Results shown represent the average ± SEM from a minimum of two experiments, each of which was repeated with at least four replicates. Single asterisk indicates statistical significance of the individual mutants by comparison to the LAC parent strain (*P* < 0.05). Double asterisks indicate significance of the double mutant relative to the appropriate isogenic single mutant (*P* < 0.05). (B) Biofilm formation was assessed in LAC and its regulatory mutants as a function of the relative capacity to produce extracellular proteases. Protease positive refers to strains with the capacity to produce all extracellular proteases. Protease deficient refers to strains unable to produce aureolysin, SspA, SspB, and ScpA. Results shown represent the average ± SEM from a minimum of two experiments, each of which was repeated with at least six replicates. Asterisk indicates statistical significance of protease-deficient derivatives relative to the respective isogenic protease-positive strains (*P* < 0.05).

Mutation of *sarA* is known to result in greatly increased levels of extracellular protease production, and this has been directly correlated with the reduced capacity of a LAC *sarA* mutant to form a biofilm under both in vitro and in vivo conditions (Tsang et al. 2008; Zielinska et al. 2011, 2012). To assess relative levels of protease activity, we used the Protease Fluorescent Detection Kit (Sigma Chemical Co.) which employs a fluorescein isothiocyanate (FITC)-casein substrate. These experiments confirmed that mutation of *rsbU*, *sigB,* and *codY*, all of which had a reduced capacity to form a biofilm (Fig.1), also resulted in increased protease production in LAC (Fig.5). Additionally, by comparison to the isogenic mutants, limiting the production of extracellular proteases by mutagenesis of the genes encoding aureolysin, SspA, SspB, and ScpA enhanced biofilm formation in all of these mutants (Fig.5). Collectively, these results strongly support the hypothesis that increased protease production makes a significant contribution to the biofilm-deficient phenotype of *sarA*, *codY*, *rsbU,* and *sigB* mutants.

These results demonstrate a correlation between increased protease production and decreased biofilm in all biofilm-deficient mutants other than the *atl* mutant. It has been suggested that the autolysin encoded by *atl* facilitates the initial attachment stage of biofilm formation both directly by functioning as an adhesin, and indirectly by promoting the release of eDNA, with FnbA and FnbB subsequently being required for biofilm maturation, particularly in methicillin-resistant *S. aureus* (MRSA) strains (Houston et al. 2011). The fibronectin-binding proteins are recognized targets of protease-mediated degradation in *sarA* mutants (Karlsson et al. 2001; Mrak et al. 2012), but in this scenario relative levels of protease production would presumably be irrelevant owing to the reduced capacity of an *atl* mutant to initiate the process of biofilm formation. Even so, increased protease production would be relevant in an *atl/sarA* mutant because it would limit FnbA/FnbB-associated accumulation. This provides a likely explanation for why concomitant mutation of *sarA* further reduced biofilm formation in the *atl* mutant, particularly since protease activity was increased in the *atl/sarA* mutant by comparison to both the isogenic *atl* mutant and LAC itself (Fig5). Mutation of *sarA* has also been shown to result in reduced accumulation of Atl itself owing to protease-mediated degradation (Zielinska et al. 2012), but this is unlikely to play a primary role in defining the biofilm-deficient phenotype of an *atl/sarA* mutant because, if it did, mutation of *sarA* would not further decrease biofilm formation by comparison to an *atl* mutant (Fig.3).

Mutation of *mgrA* or *fur* also had no impact on protease production by comparison to LAC (Fig.5). However, limiting the production of proteases did enhance biofilm formation in the *mgrA* mutant. To the extent that concomitant mutation of *sarA* in the *mgrA* mutant also resulted in increased protease production by comparison to the isogenic *mgrA* mutant, this also provides a likely explanation for why concomitant mutation of *sarA* reversed the increased capacity of the *mgrA* mutant to form a biofilm (Fig.3). Mutation of *sarA* also reversed the increased biofilm formation observed in the LAC *fur* mutant (Fig.3), and resulted in a statistically significant increase in protease production, but the relative capacity of the *fur* mutant to form a biofilm under our assay conditions was not altered to a statistically significant extent by limiting the production of extracellular proteases (Fig.5). This suggests the involvement of other factors in defining the enhanced biofilm phenotype of a LAC *fur* mutant.

Mutation of *fur* in the commonly studied strain Newman, which notably does not produce surface-anchored fibronectin-binding proteins (Grundmeier et al. 2004), also enhanced biofilm formation under iron-limiting conditions, but only during the early stages of biofilm formation (Johnson et al. 2005). The mechanistic basis for these phenotypes was not explained although it appeared to be independent of any impact on accumulation of the PIA. To the extent that our assays were done using a nutrient-rich medium, and the results assayed after a 24 h incubation period, the increased capacity of the LAC *fur* mutant to form a biofilm under our assay conditions is in contrast to this report, although we did confirm that mutation of *fur* had no detectable impact on the accumulation of PIA in LAC (see below). A previous paper described a number of conserved surface Fur-regulated proteins (Frp) and suggested that at least two of these (FrpA and FrpB) are involved in the initial attachment stage of biofilm formation (Morrissey et al. 2002). Since Fur represses the production of these proteins in the presence of iron, one could hypothesize that mutation of *fur* would result in an increase in Frp expression and consequently biofilm formation. Eap and Emp have also been implicated in biofilm formation (Palma et al. 1999; Hussain et al. 2001), but both of these are positively regulated by Fur at least under iron-restricted conditions, thus suggesting that they would be produced in decreased amounts in a *fur* mutant (Johnson et al. 2008).

### Impact of extracellular nuclease production on biofilm formation

**Figure 6:**
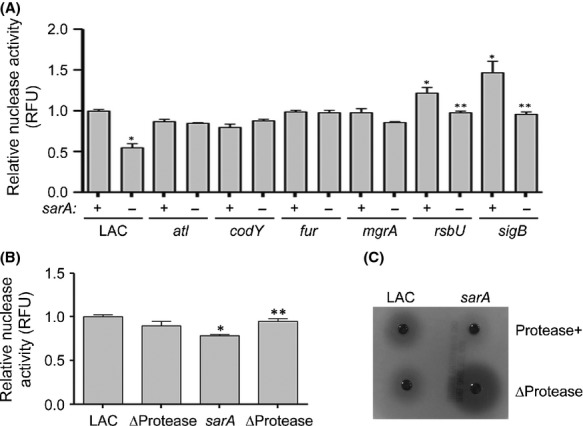
Impact of extracellular nucleases in LAC. (A) Total nuclease activity was assessed in LAC regulatory mutants with (−) and without (+) concomitant mutation of *sarA* using a FRET-based assay. Results shown represent the average ± SEM from a minimum of two experiments, each of which was repeated with at least four replicates. Single asterisk indicates statistical significance of the individual mutants by comparison to the LAC parent strain (*P* < 0.05). Double asterisks indicate significance of the double mutant relative to the corresponding isogenic single mutant (*P* < 0.05). (B) Relative levels of nuclease activity were assessed as above, but as a function of the production of extracellular proteases. Single asterisk indicates statistical significance of the individual mutants by comparison to the LAC parent strain (*P* < 0.05). Double asterisks indicate significance of the *sarA/*ΔProtease mutant relative to the corresponding isogenic *sarA* mutant (*P* < 0.05). (C) For comparison, relative levels of nuclease activity were also assessed using DNase Agar assay.

The results discussed above demonstrate an important role for extracellular proteases in defining the biofilm phenotype of most but not all of the regulatory mutants we examined. To determine whether the production of extracellular nucleases may account for at least some of these exceptions, we also assessed nuclease activity using a FRET-based assay (Kiedrowski et al. 2014). The only mutants that exhibited a significant increase in nuclease activity were the *rsbU* and *sigB* mutants (Fig.6). This raises the possibility that this also contributes to the biofilm-deficient phenotype of these mutants. However, limiting protease production enhanced biofilm formation in both of these mutants (Fig.3). Additionally, mutation of *sarA* reversed the increase in nuclease production in the *rsbU* and *sigB* mutants (Fig.6), and this was correlated with a further decrease, rather than an increase, in biofilm formation (Fig.1). Indeed, mutation of *sarA* resulted in reduced nuclease activity in LAC, and we confirmed that this is reversed by eliminating the ability of *sarA* mutants to produce extracellular proteases (Fig.6), thus demonstrating that the impact of *sarA* on nuclease activity in LAC occurs via an indirect mechanism involving protease-mediated degradation. More importantly, biofilm formation was increased in a protease-deficient *sarA* mutant (Fig.5) despite the increase in nuclease activity (Fig.6). Taken together, these results suggest that the increased production of proteases plays the more important role, by comparison to the increased production of extracellular nucleases, in defining the biofilm-deficient phenotype of *sarA*, *rsbU* and *sigB* mutants.

Nuclease activity was unchanged in *atl*, *fur*, or *mgrA* mutants (Fig.6), but this does not preclude a role for eDNA in at least some of these mutants. In fact, in some cases the more relevant consideration may be that nuclease production was not increased. For instance, Trotonda et al. (2008) proposed that mutation of *mgrA* increases expression of *cidA* and decreases expression of *lrgAB*, the combined result of which is increased autolysis and increased availability of eDNA, and under these circumstances it is potentially important that mutation of *mgrA* did not result in increased nuclease activity. This same report also found that mutation of *sarA* reversed the increased biofilm formation observed in the *mgrA* mutant, but it was concluded that this was independent of the increased production of aureolysin or SspA (Trotonda et al. 2008). However, this report examined the impact of these proteases independently of each other, and our studies confirm that the impact of *sarA* on biofilm formation involves the increased production of multiple proteases (Loughran et al. 2014). In the case of the *atl* mutant, nuclease production would presumably be irrelevant owing to the reduced availability of eDNA as detailed above. In the case of *fur*, there is a report demonstrating that the mutation of *fur* represses expression of the genes encoding extracellular nucleases (Johnson et al. 2011), and this would presumably promote biofilm formation. Mutation of *fur* did enhance biofilm formation in LAC under the experimental conditions we employed, but the fact that nuclease production was unchanged in the LAC *fur* mutant suggests that extracellular nucleases cannot account for this phenotype. It is also important to recognize that the impact of *fur* on *S. aureus* phenotypes is dependent to a large extent on iron availability (Morrissey et al. 2002; Johnson et al. 2011), and we have not yet addressed this issue.

### Impact of PIA production on biofilm formation

**Figure 7:**
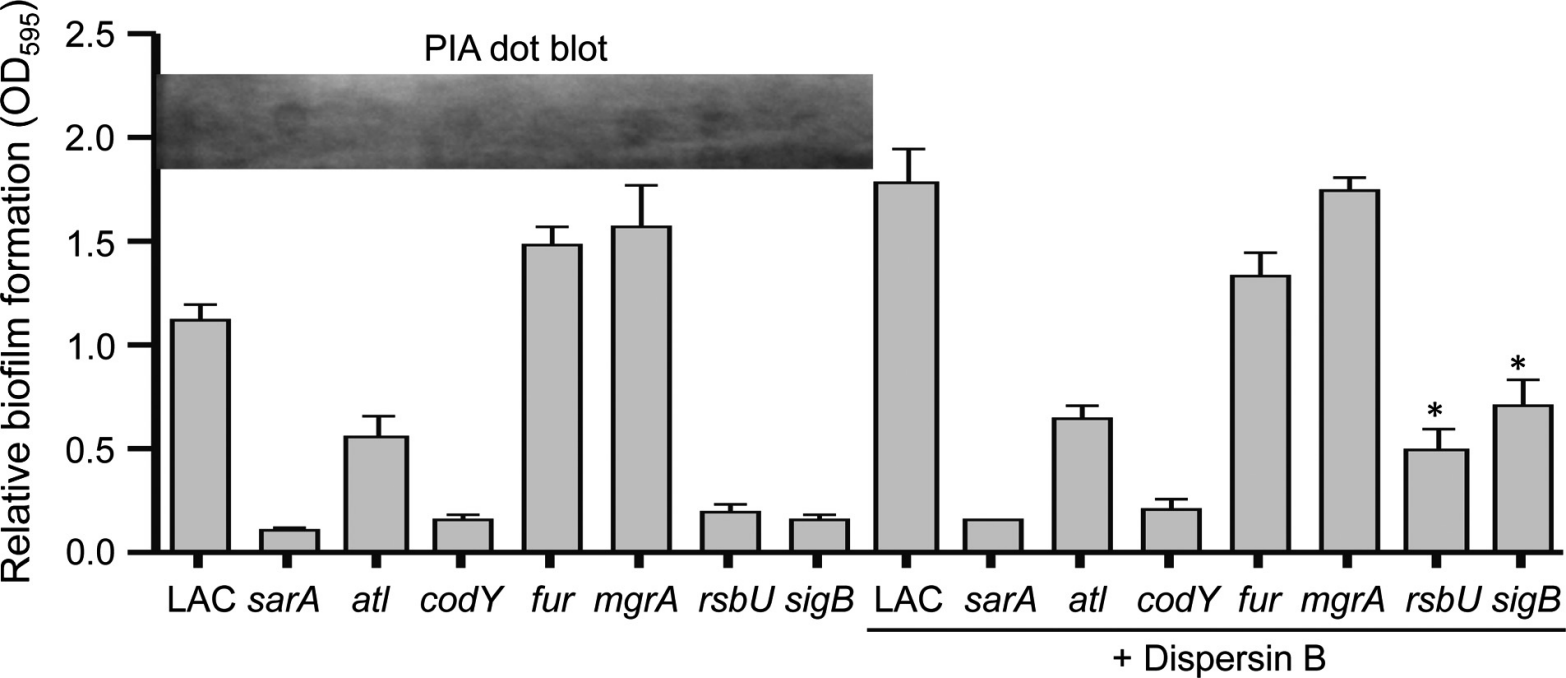
Impact of PIA in LAC. Biofilm formation was assessed using a microtiter plate assay with and without the addition of Dispersin B. Results shown represent the average ± SEM from a minimum of two experiments, each of which was repeated with at least three replicates. Asterisks indicate mutants in which the addition of Dispersin B had a statistically significant impact by comparison to the same strain in the absence of Dispersin B (*P* < 0.05). Inset illustrates levels of PIA production in LAC and its indicated isogenic mutants in the absence of Dispersin B.

We next assessed whether production of the PIA (also known as poly-*N*-acetyl-*β*-(1–6)-glucosamine or PNAG) might contribute to the biofilm phenotypes we observed. This was complicated by the fact that we could not detect appreciable amounts of PIA in immunoblots with LAC or any of its mutants (Fig.7). As an alternative approach, we examined the impact of Dispersin B, a known inhibitor of PIA-mediated biofilm formation (Donelli et al. 2007; Sugimoto et al. 2013). The only strains in which Dispersin B had a significant impact were the *rsbU* and *sigB* mutants, and in both cases biofilm formation was increased rather than decreased in the presence of Dispersin B (Fig.7). Although the reasons PIA would limit biofilm formation remain unclear, we have observed this phenotype before (Loughran et al. 2014), and it is generally consistent with the suggestion that biofilm formation in *S. aureus*, particularly in MRSA strains such as LAC, is largely independent of PIA production (O'Neill et al. 2008; Pozzi et al. 2012). Indeed, one possible explanation for the increase in biofilm formation observed in LAC in the presence of Dispersin B is that the abundance of PIA, or other exopolysaccharides, was reduced to the point of increasing the exposure of surface proteins that promote biofilm formation.

### Impact of select mutations in UAMS-1

The results discussed above are consistent with the following conclusions: (1) *sarA* plays a primary role in *S. aureus* biofilm formation in the USA300 strain LAC owing to its ability to repress the production of extracellular proteases; (2) protease production also plays an important role in limiting biofilm formation in *rsbU*, *sigB,* and *codY* mutants; (3) in those cases in which this is not the case, including those in which a mutation is associated with an enhanced capacity to form a biofilm, the impact of *sarA* on biofilm formation is epistatic to the impact of these other regulatory loci. However, these studies were limited to the MRSA strain LAC, and as noted above, it has been suggested that the mechanism of biofilm formation differs as a function of methicillin resistance (Houston et al. 2011). We therefore examined the impact of a subset of these mutations in the MSSA strain UAMS-1. These studies were limited by difficulties in transducing mutations from the JE2 NTML derivatives, or their LAC transductants, into UAMS-1, but we had previously generated *codY*, *mgrA*, and *sigB* mutations in both UAMS-1 and its isogenic *sarA* mutant. Mutation of *sigB* and *mgrA* was found to have the same impact on biofilm formation in UAMS-1 and LAC (i.e., decreased in the former and increased in the latter). Similarly, Bose et al. (2012) previously demonstrated that mutation of *atl* limits biofilm formation in UAMS-1. Thus, the same general trends were observed in the context of these loci in both UAMS-1 and LAC.

**Figure 8:**
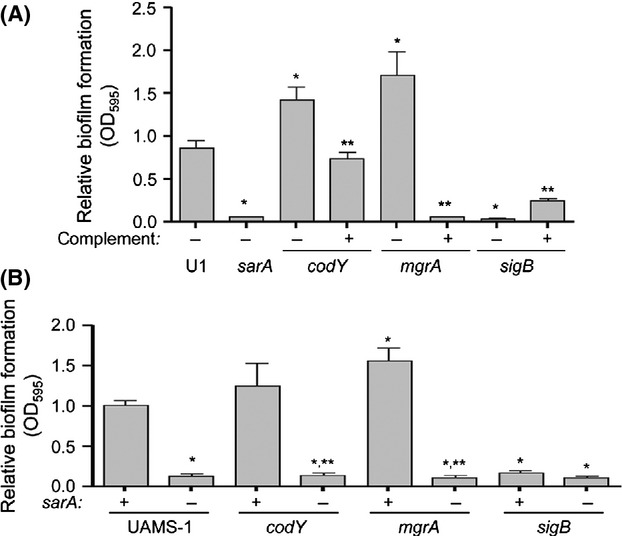
Relative impact of *sarA* versus other regulatory loci in UAMS-1. (A) Biofilm formation was assessed in each regulatory mutant found to have a significant impact on biofilm formation with (+) and without (−) plasmid-based genetic complementation. Results shown represent the average ± SEM from a minimum of three experiments, each of which was repeated with at least three replicates. Single asterisk indicates that the results observed with the indicated mutant were significantly different from those observed with the UAMS-1 (U1) parent strain (*P* < 0.05). Double asterisks indicate that the results observed with the complemented strain were significantly different by comparison to those observed with the uncomplemented isogenic mutant (*P* < 0.05). (B) Biofilm formation was assessed in each regulatory mutant found to have a significant impact on biofilm formation with (−) and without (+) concomitant mutation of *sarA*. Single asterisk indicates statistical significance of the individual mutants by comparison to the UAMS-1 parent strain (*P* < 0.05). Double asterisks indicate significance of the double mutant relative to the corresponding isogenic single mutant (*P* < 0.05).

**Figure 9:**
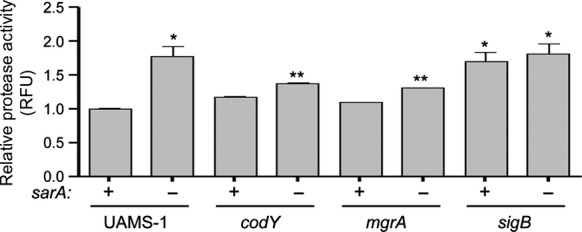
Impact of extracellular proteases in UAMS-1. Total protease activity was assessed in UAMS-1 mutants with (−) and without (+) concomitant mutation of *sarA*. Results shown represent the average ± SEM from a minimum of two experiments, each of which was repeated with at least four replicates. Single asterisk indicates statistical significance by comparison to the UAMS-1 parent strain (*P* < 0.05). Double asterisks indicate significance of the double mutant relative to the corresponding isogenic single mutant (*P* < 0.05).

**Figure 10:**
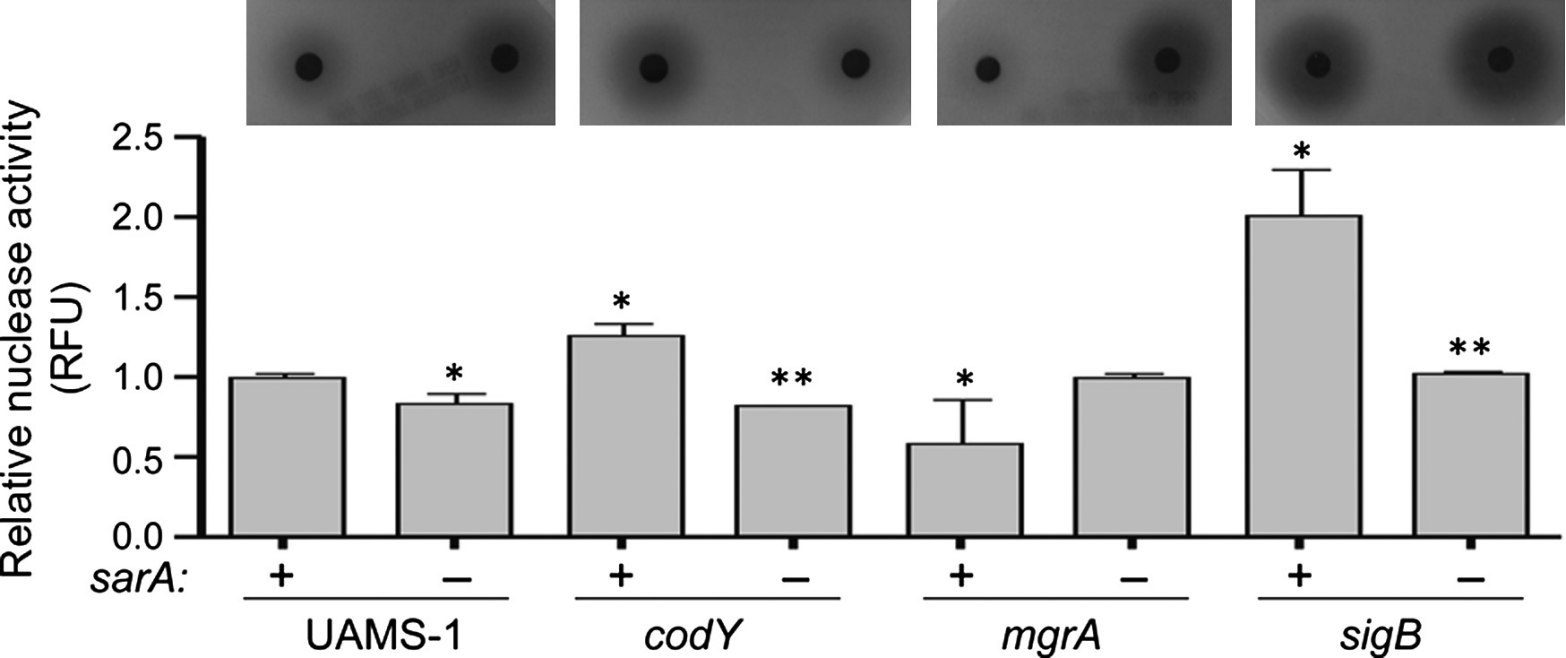
Impact of extracellular nucleases in UAMS-1. Total nuclease activity was assessed in UAMS-1 and its isogenic mutants with (−) and without (+) concomitant mutation of *sarA* using a FRET-based assay. Results shown represent the average ± SEM from a minimum of two experiments, each of which was repeated with at least four replicates. Single asterisk indicates statistical significance by comparison to the isogenic parent strain (*P* < 0.05). Double asterisks indicate significance of the double mutant relative to the respective isogenic single mutant (*P* < 0.05). Inset illustrates results observed using DNase agar.

In contrast, mutation of *codY* in UAMS-1 increased rather than decreased biofilm formation (Fig.8). Our results are consistent with a previous report demonstrating that mutation of *codY* increased biofilm formation in UAMS-1, a phenotype that was attributed to the increased production of PIA (Majerczyk et al. 2008). This possibility is consistent with the observation that protease activity was not significantly increased in a UAMS-1 *codY* mutant (Fig.9). Mutation of *codY* in UAMS-1 did result in increased nuclease activity (Fig.10), which is interesting given that it had the opposite effect in LAC, but this is unlikely to be important in that a UAMS-1 *codY* mutant had an increased capacity to form a biofilm. Additionally, Dispersin B limited biofilm formation not only in a UAMS-1 *codY* mutant, but also in the isogenic *mgrA* mutant (Fig.1). This implicates PIA production in the biofilm phenotype of both of these mutants. This was confirmed by demonstrating the PIA production was increased in both UAMS-1 *codY* and *mgrA* mutants (Fig.1).

**Figure 11:**
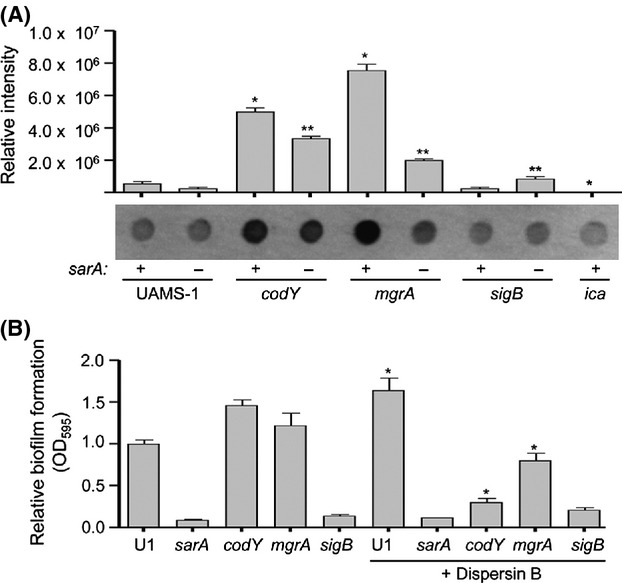
Impact of PIA in UAMS-1. (A) PIA production as assessed by dot blot. Graph illustrates quantitative results obtained from three independent blots, with a representative dot blot shown below the graph. Single asterisk indicates statistical significance by comparison to the isogenic UAMS-1 (U1) parent strain (*P* < 0.05). Double asterisks indicate significance of the double mutant relative to the appropriate isogenic single mutant (*P* < 0.05). (B) Biofilm formation was assessed with and without Dispersin B. Results shown represent the average ± SEM from a minimum of two experiments, each of which was repeated with at least three replicates. Asterisk indicates mutants in which the addition of Dispersin B had a statistically significant impact by comparison to the same strain in the absence of Dispersin B (*P* < 0.05).

**Figure 12:**
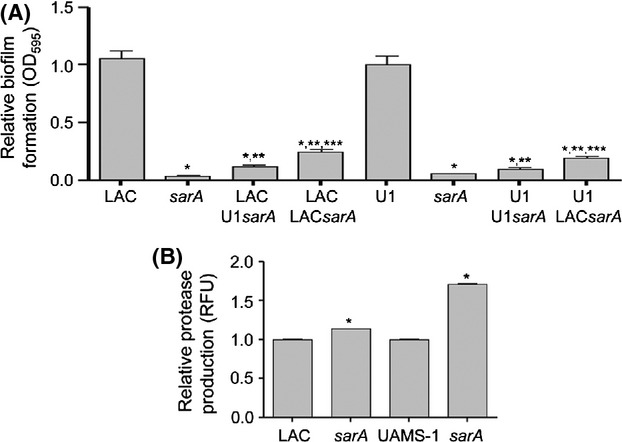
Impact of *sarA* mutants on wild-type biofilm phenotypes. (A) Biofilm formation was assessed in LAC or UAMS-1 (U1) after coculture with the indicated *sarA* mutants, with each parent strain and its isogenic *sarA* mutant included as controls. Results shown represent the average ± SEM from a minimum of three experiments, each of which was repeated with at least three replicates. Single asterisk indicates statistical significance by comparison to the respective parent strain (*P* < 0.05). Double asterisks indicate statistical significance by comparison to the isogenic *sarA* mutant (*P* < 0.05). Triple asterisks indicate a statistically significant difference between results observed with the LAC *sarA* mutant by comparison to the UAMS-1 *sarA* mutant (*P* < 0.05). (B) Total protease activity was assessed in LAC, its isogenic *sarA* mutant, UAMS-1, and its isogenic *sarA* mutant. WT parent strains, LAC, and UAMS-1, were set to 1.0 with the respective *sarA* mutants set relative to that. Results shown represent the average ± SEM from a minimum of two experiments, each of which was repeated with at least four replicates. Single asterisk indicates statistical significance by comparison to the respective parent strain (*P* < 0.05).

Concomitant mutation of *sarA* reversed both the increased production of PIA (Fig.11) and increased the capacity of *codY* and *mgrA* mutants to form a biofilm (Fig.8). This suggests a cause-and-effect relationship. However, the limitation of PIA production observed in UAMS-1 *codY/sarA* and *mgrA/sarA* mutants relative to their isogenic *codY* and *mgrA* mutants was modest by comparison to the biofilm phenotypes of these mutants, and the level of PIA production was comparable in UAMS-1 and its *sarA* mutant (Fig.11) despite their dramatically different biofilm phenotypes (Fig.8). These results confirm that *sarA* plays a defining role in both the MRSA strain LAC and the MSSA strain UAMS-1 and that, in both strains, the primary phenotypic impact of *sarA* is a function of its impact on the production of extracellular proteases. Further support for this hypothesis comes from the observations that biofilm formation was limited in mixed culture assays consisting of either LAC or UAMS-1 together with *sarA* mutants generated in either strain (Fig.12). Additionally, coculture with the LAC *sarA* mutant limited biofilm formation in both LAC and UAMS-1 to a lesser degree than co-culture with the UAMS-1 *sarA* mutant, which is consistent with the observation that mutation of *sarA* resulted in a greater increase in protease production in UAMS-1 than in LAC (Fig.12).

**Figure 13:**
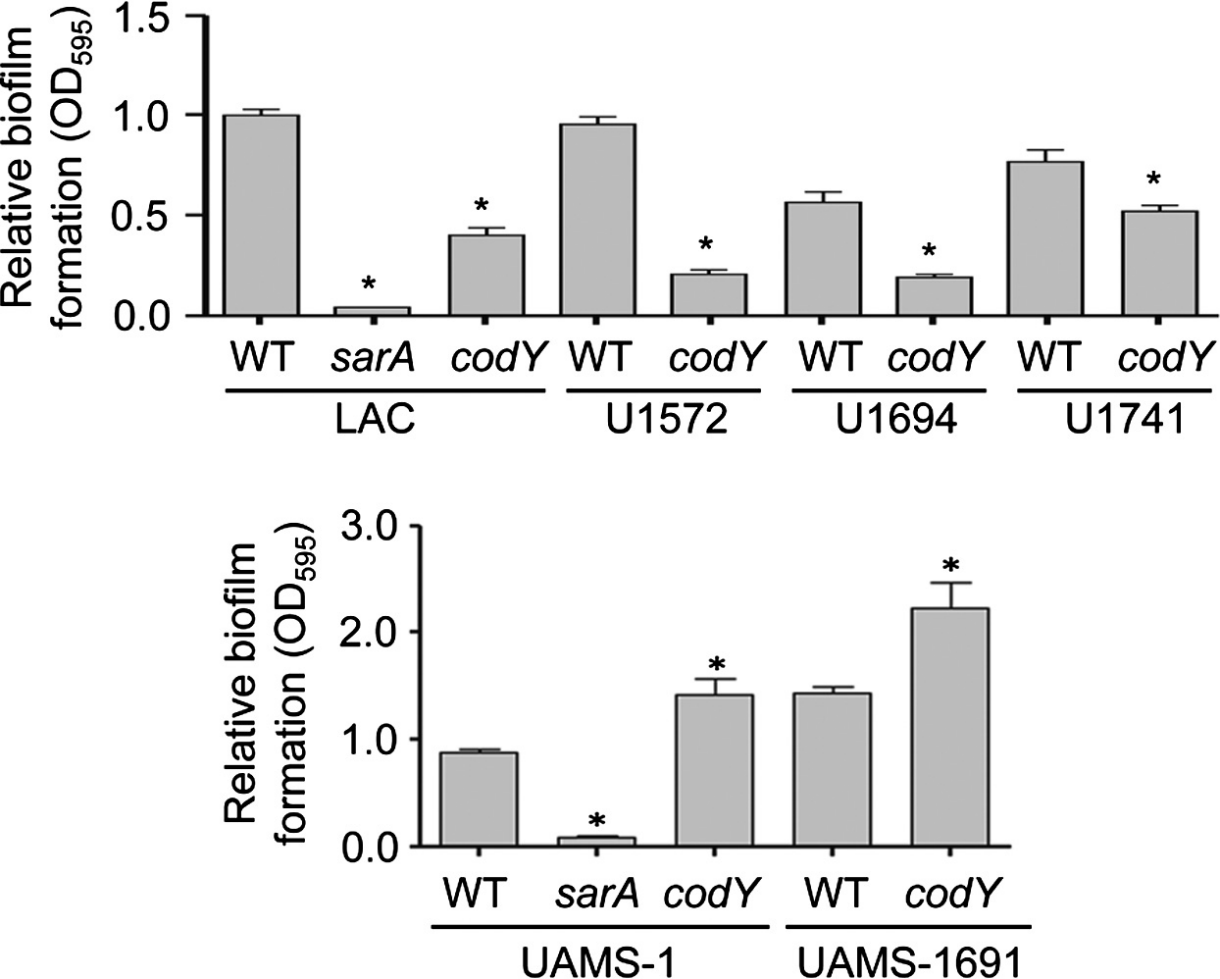
Strain-dependent impact of *codY* on biofilm formation. The impact of mutating *codY* on biofilm formation was assessed as a function of methicillin resistance status, with the strains shown in the upper panel being MRSA isolates, and those shown in the lower panel being MSSA isolates. Results shown represent the average ± SEM from a minimum of two experiments, each of which was repeated with at least six replicates. Asterisk indicates statistical significance by comparison to the respective parent strain (*P* < 0.05).

These results provide further support for the importance of limiting protease production as a means of promoting biofilm formation in both the MRSA strain LAC and the MSSA strain UAMS-1. Nevertheless, they also reveal an important strain-dependent difference in the context of *codY*. To investigate this further, we transduced the *codY* mutation into additional clinical isolates and examined the impact on biofilm formation. We were limited in this case, owing to antibiotic resistance issues in the targeted clinical isolates, but we were able to successfully transduce this mutation into three additional MRSA strains and one additional MSSA strain. Biofilm formation was reduced in the *codY* mutants generated in all three MRSA strains and increased in the additional MSSA strain (Fig.13). This suggests that the differential impact of mutating *codY* on biofilm formation may be directly correlated with methicillin resistance status, and that this is likely a function of the impact of mutating *codY* on the production of PIA. However, there are contradictory reports in the literature regarding the impact of *codY* on biofilm formation, with Majerczyk et al. (2008) concluding as we observed that mutation of *codY* in UAMS-1 enhances biofilm formation, and Tu Quoc et al. (2007) concluding that mutation of *codY* in the *S. aureus* strain S30 has the opposite effect, and both of these are methicillin-sensitive strains. Thus, this potential correlation warrants further study and is an area of active investigation in our laboratory.

## Conclusion

In summary, the only mutation we identified that significantly impacts biofilm formation in a manner that could not be correlated with protease or PIA production is the LAC *fur* mutant, and even in this case mutation of *sarA* reversed the phenotypic impact of mutating *fur*. Thus, our results confirm the primary importance of *sarA* in the context of biofilm-associated *S. aureus* infections. Based on this, we believe the results we report strongly support the hypothesis that inhibitors of *sarA*-mediated regulation would have tremendous potential in the context of overcoming the pathology and therapeutic recalcitrance of these infections owing to their ability to increase the production of extracellular proteases, and that this would be true irrespective of the functional status of other *S. aureus* regulatory loci.

## Supporting Information

Figure S1 Verification of LAC mutants and biofilm phenotypes in select mutants generated in JE2 and LAC. (A) Comparative analysis of EcoRI-digested genomic DNA from strains derived from JE2 or LAC. Arrow indicates the small plasmid present in our derivative of LAC but absent in JE2 and its NTML derivatives. (B) Biofilm formation was assessed using a microtiter plate assay. Results shown represent the average ± standard error of the mean (SEM) from a minimum of three experiments, each of which was repeated with at least six replicates. Results with LAC were set to 1.0. The results observed with all other strains, including JE2, shown relative to this value. Asterisk indicates significance by comparison to the corresponding parent strain (*P* < 0.05).
