# Supplementary figures and images for: Comparative impact of diverse regulatory loci on Staphylococcus aureus biofilm formation
Source: Microbiologyopen. 2015 Mar 21;4(3):436–51. doi: 10.1002/mbo3.250 (PMC4475386; doi:10.1002/mbo3.250)

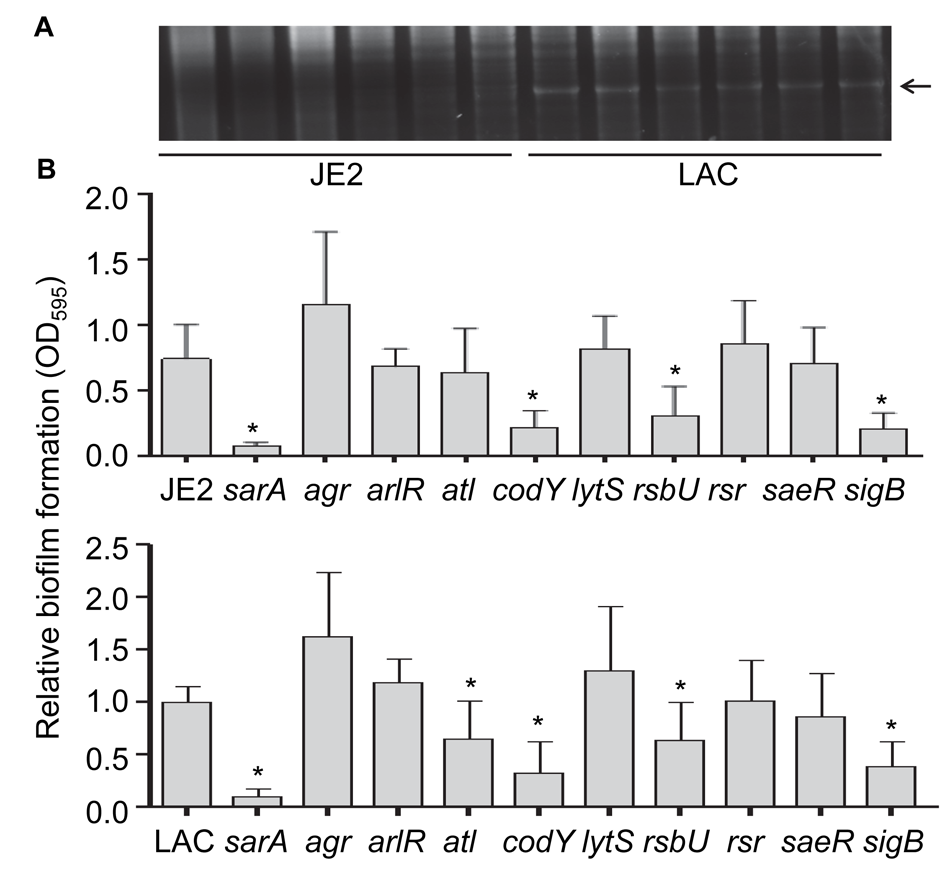

Supplement: Figure S1 — Verification of LAC mutants and biofilm phenotypes in select mutants generated in JE2 and LAC. (A) Comparative analysis of EcoRI-digested genomic DNA from strains derived from JE2 or LAC. Arrow indicates the small plasmid present in our derivative of LAC but absent in JE2 and its NTML derivatives. (B) Biofilm formation was assessed using a microtiter plate assay. Results shown represent the average ± standard error of the mean (SEM) from a minimum of three experiments, each of which was repeated with at least six replicates. Results with LAC were set to 1.0. The results observed with all other strains, including JE2, shown relative to this value. Asterisk indicates significance by comparison to the corresponding parent strain (P < 0.05). [file mbo30004-0436-sd1.tif]
